# The success rates and outcomes of mandibular third molar coronectomy: 167 cases

**DOI:** 10.1007/s10006-024-01244-z

**Published:** 2024-04-03

**Authors:** Sylwia Maria Nowak, Jessie Justice, Aneesah Aslam, Mohamed Imran Suida

**Affiliations:** 1 Oral Surgery Department, Leeds Dental Institute, Leeds Teaching Hospitals NHS Trust, Leeds, UK; 2 Oral and Maxillofacial Department, Bradford Teaching Hospitals NHS Trust, Bradford, UK; 3 Facial Department, Mid-Yorkshire Teaching Hospitals NHS Trust, Wakefield, UK; 4 Oral Surgery Department, Cardale and Huddersfield NHS Trust, Huddersfield, UK

**Keywords:** Coronectomy, Wisdom tooth, Third molar, Success rates, Complications, Nerve injury

## Abstract

**Purpose:**

The purpose of this study was to assess success rates and to report complications of coronectomy of mandibular third molars (M3M), including intra-operative failure, pain, infection, dry socket, inferior dental alveolar (IAN) and lingual nerve (LN) injuries and re-operation rates.

**Methods:**

Retrospective analysis of 167 coronectomies completed between January 2017 to December 2022 was undertaken.

**Results:**

The success of coronectomy was 93%. Intra-operative failure was reported to be 3.6% (*n* = 8). Complications accounted for pain (15%, *n* = 24), infection (9%, *n* = 15) and dry socket (3.6%, *n* = 6). Three patients required removal of M3M root at 3 months (*n* = 2) and 24 months (*n* = 1), accounting for 1.8% re-operation rate. A total of number of patients who suffered a nerve injury was 12; three of these were permanent (LN – 1.2%, *n* = 2; IAN – 0.6%, *n* = 1), nine were temporary (IAN – 1.2%; *n* = 2, LN – 2.4%; *n* = 4; site not specified – 1.8%, *n* = 3). No patients with intra-operative failure and re-operation suffered IAN or LN injury post-operatively.

**Conclusion:**

Coronectomy offers a successful strategy for management of high risk M3M. The treatment outcomes can be improved with careful case selection and adjusting surgical technique, including assessment of root morphology, incomplete crown sectioning technique and avoidance of lingual retraction. Reporting of coronectomy success as a factor of surgical outcome, presence or absence of permanent IAN injury, persistent symptoms or any other long-standing complications (such as LN injury), and the need for re-operation accounting for root migration status may be a useful tool to measure coronectomy outcomes.

## Introduction

Coronectomy is an alternative surgical technique for the management of high risk mandibular third molars (M3M). It involves removal of the coronal portion of the tooth and the retention of the roots with the aim to reduce the risk of injury to the inferior dental alveolar nerve (IAN) [1].

High risk M3M are identified on an orthopantomogram (OPT) by radiological signs described by Rood & Shehab [2] such as darkening, narrowing or deflection of the roots, bifid root apex, narrowing or diversion of the IAN canal and a loss or interruption of superior cortical outline of the IAN. The presence of juxta-apical radiolucency is considered as an additional high risk sign detectable on an OPT [3].

Dental cone beam computer tomography (CBCT) further assists with determining the true relation of M3M to the IAN canal, as 30% of high risk M3M seen on a OPT are found distant on a CBCT [4]. CBCT has been found to significantly modify the decision making in treatment planning between surgical removal and coronectomy compared with OPT alone [5]. Features on a CBCT suggestive of high risk relationship include interruption of cortical line of IAN canal more than 3 mm, deformation of IAN canal, bifid IAN canal in contact with tooth, M3M root perforation by the IAN canal, inter-radicular or lingual position of IAN canal [4, 6].

Imaging strategies are the basis of surgical treatment planning but have low value in prediction of post-operative IAN injury. Thus, M3M treatment options and their associated risks versus benefits should be discussed with the patient carefully. The Royal College of Surgeons of England ‘Parameters of Care’ [7] set out selection criteria for coronectomy, including tooth-related, medical and social history factors (Table 1). These should be established during the initial consultation prior coronectomy planning.

Coronectomy may reduce the risk of IAN injury by 84% [8], however, it carries risks which may negatively influence the outcome of the treatment. Complications include intra-operative failure requiring removal at the time of surgery, dry socket, early and late infection and root migration. Re-operation may be necessary to manage some of these complications. Intra-operative removal of roots and early recurrent post-operative infections result in the need for M3M root removal, increasing the risk of IAN injury to the risk predicted of that of surgical extraction. Although most root migration occurs within the first 6 months, it is seen to occur within the first 2 months post treatment [9]. Late infections requiring surgical intervention where root migration is noted may have a negligible risk to the IAN, thus, can be considered as a favourable outcome.

Studies in the literature describe outcomes of coronectomy, whereas success rates are generally not reported. We define a successful outcome as a M3M surgery where the crown of M3M was removed such that the roots remain immobile in situ without causing any permanent injury to IAN, and the patient remains asymptomatic following the healing period, and/or when the patient did not suffer any other long-standing complications that may or may not impair their quality of life.

The purpose of this study was to assess success rates and to report complications of coronectomy in our 6-year experience.

**Table 1:** Contraindications for coronectomy in accordance with Royal College of Surgeons England (Parameters of Care, 2020)

Tooth factors	Medical factors	Social factors
Non-vital pulp Caries with pulpal involvement Tooth mobility Apical disease Associated with cystic tissuing unlikely to resolve if root left in situ Associated with tumors	Immunocompromised patient Head and neck radiotherapy (previous, planned) Neuromuscular disorders Diabetes mellitus (poorly controlled)	Unable to return for treatment with ease

## Materials and methods

The retrospective data collection was approved by the Mid-Yorkshire Teaching NHS Trust Audit Committee. Patients included in the study were referred to the Mid-Yorkshire Teaching NHS Trust from primary and secondary sectors for management of wisdom teeth. Patients who underwent coronectomy of M3Ms between January 2017 and December 2022 were identified through the National Health Service Coding System (Coronectomy, F09.6). Non-M3M coronectomies were excluded. The data was collected from electronic patient records to review clinical letters and operation notes and PACS Xero System to review radiographic records (OPT and CBCT). No ethical approval was required.

Treatment was performed under local anaesthetic, intravenous sedation, or general anaesthetic by surgeons of varying grade of surgical experience, including junior and senior specialty trainees, specialty dentists, Oral Surgery specialists and consultants in Oral and Maxillofacial Surgery. The procedure calibration could not be assured.

The secondary outcomes of the study were to assess:

- Patients’ demographics.
- Imaging assessment strategies.
- Rate of post-operative complications including pain, dry socket, infection, nerve injury and other adverse permanent complications.
- Follow-up protocols.

Acute complications were considered when patient reported to the acute team by telephone contact or by presenting to accident and emergency department. Pain was patient reported and only accounted as a complication when it was not self-managed with over-the-counter analgesia.

## Results

### Patient demographics

Patient characteristics are described in Table 2. In total, 143 patients underwent 167 coronectomies; 60% had a single coronectomy (*n* = 101), whereas 40% had bilateral coronectomies (*n* = 66). Majority of patients were female (78%, *n* = 131). Patients were aged from 17 to 91 years, with a mean age of 32. No relevant medical history was recorded for 94% (*n* = 157). One patient had well-controlled diabetes mellitus type 2 and one was taking low-dose systemic steroids. Twenty patients were smokers.

**Table 2:** Baseline characteristics: Patient Demographics

		%	N=
Sex	Female Male	78 22	131 36
Age	32 years (17–91)		
Medical history	No relevant Steroid use Diabetic Other Not available	94 0.5 0.5 3 1	157 1 1 5 2
Social history	No relevant Smoker Alcohol > 50 units/week Not available	68 12 2 18	114 20 3 30

### Assessment strategies

#### Clinical indication

Coronectomy was undertaken for M3M affected by multiple episodes of pericoronitis in 84%, cyst or other pathology in 9%, caries in mandibular second molar (M2M) in 5%, risk of caries in M2M in 1% and caries in M3M in 1%.

Pulpal status of M3M was determined to be sound in 86% (*n* = 143), caries into outer third of dentine in 8% (*n* = 13), enamel caries in 5% (*n* = 8), internal/external resorption in 1% (*n* = 2) and one case of caries extending into outer but not into the inner third of dentine (no pulpal involvement).

The two cases of resorption were found to be successful on follow up, whereas the case of caries beyond outer third of dentine had no scheduled review but did not re-present with symptoms.

Summary of assessment strategies is presented in Table 3.

#### Radiographic assessment

An OPT was taken for 95% of cases, whereas a periapical radiograph was used for the remaining 5% of cases as the baseline radiograph. Radiographic assessment identified one or more high risk signs as described by Rodd & Shehab [2] in 95% of cases treated.

A dental CBCT was undertaken for 78% of cases (*n* = 131). Out of the remaining 22% (*n* = 35) that did not have a CBCT, 57% (*n* = 20) declined it, 3% (*n* = 1) had bony separation present on OPT (thus, CBCT was not indicated). For 40% (*n* = 14) no information was given about the CBCT.

High risk signs on CBCT were present for 92% cases (*n* = 120) and 6% (*n* = 8) of cases lacked the classic ‘high risk signs’. The risk level could not be determined for 2% (*n* = 3) due to movement artefact or scan not available on the PACS XERO system.

Over 70% of the cases had more than one high risk sign detectable on a CBCT (Table 3). The following were recorded: interruption of cortication > 3 mm (*n* = 59, 29.8%), deformation of IAN in contact with roots presenting as dumbbell (*n* = 15, 7.6%), tear drop (*n* = 23, 11.6%) or other (*n* = 38, 19.2%), lingual IAN position (*n* = 32, 16.2%), inter-radicular IAN position (*n* = 24, 12.1%) and bifid IAN canal in contact with roots (*n* = 1, 0.5%).

In the cases without the classic ‘high risk signs’, the CBCT found the presence of bony separation (*n* = 4) and altered root morphology, such as root dilaceration (*n* = 1), hooked root (*n* = 2) and root apex ankylosis (*n* = 1). In these cases, patient-led informed decision directed the treatment choice (coronectomy versus surgical removal).

**Table 3:** Clinical and radiographic assessment of M3M

		%	N=
Indication	Chronic pericoronitis Cyst/other pathology Caries in M2M Risk of caries in M2M Caries in M3M	84 9 5 1 1	141 15 9 1 1
Pulp status M3M	Sound Enamel caries Caries into outer 1/3 dentine Caries beyond outer 1/3 dentine Internal/external resorption	86 5 8 0.5 1	143 8 13 1 2
Tooth Angulation	Vertical Mesio-angular Disto-angular Horizontal Atypical	23 29 30 16 2	37 48 50 25 3
Imaging	OPT Periapical CBCT	95 5 78	159 4 131
CBCT signs	Interruption of cortication > 3 mm	29.8	59
	Deformation of IAN canal:		
	a) Dumbbell b) Teardrop c) Other Lingual IAN position Inter-radicular IAN position Bifid IAN position Complex root morphology (dilacerated/hooked/ankylosed)	7.6 11.6 19.2 16.2 12.1 0.5 2.5	15 23 38 32 24 1 4

### Treatment

Coronectomy was undertaken under general anaesthetic (*n* = 133, 79%), local anaesthetic (*n* = 25, 15%) and operator-led intravenous sedation with midazolam (*n* = 10, 6%). Majority of bilateral coronectomies were undertaken under general anaesthetic (97%), with only 2 patients having bilateral coronectomy under local anaesthetic over 2 appointments.

Most surgeries were undertaken by Specialist Oral Surgeons (39%), followed by Specialty Trainees in Oral Surgery or Oral and Maxillofacial Surgery (26%). Consultants performed 15% of cases (Table 4).

**Table 4:** Treatment details

		%	N=
Type of Anaesthesia	LA IVS GA	15 6 79	25 10 133
Operator Grade	Dental Core Trainee Specialty Trainee Specialty Dentist Specialist Oral Surgeon Consultant Unknown	6.5 26 13 39 15 0.5	11 42 20 64 23 7

### Post operative complications

Summary of post-operative complications is listed in Table 5.

**Table 5:** Summary of post-operative complications

	%	N=
Intra-operative failure	3.6	6
Pain Infection Dry socket	14 9 3.6	24 15 6
Nerve injury		
*IAN temporary* *IAN permanent*	1.2 0.6	2 1
*LN temporary* *LN permanent*	2.4 1.2	4 2
*Site not specified - temporary*	1.8	3
Re-operation (total) *Root migration - absent* *Root migration - present*	1.8 1.2 0.6	3 2 1

#### Intra-operative complications

A total of 9 intra-operative complications were reported (5.3%), including: intra-operative failure (*n* = 8) and burn to lip (*n* = 1).

**Fig. 1:**
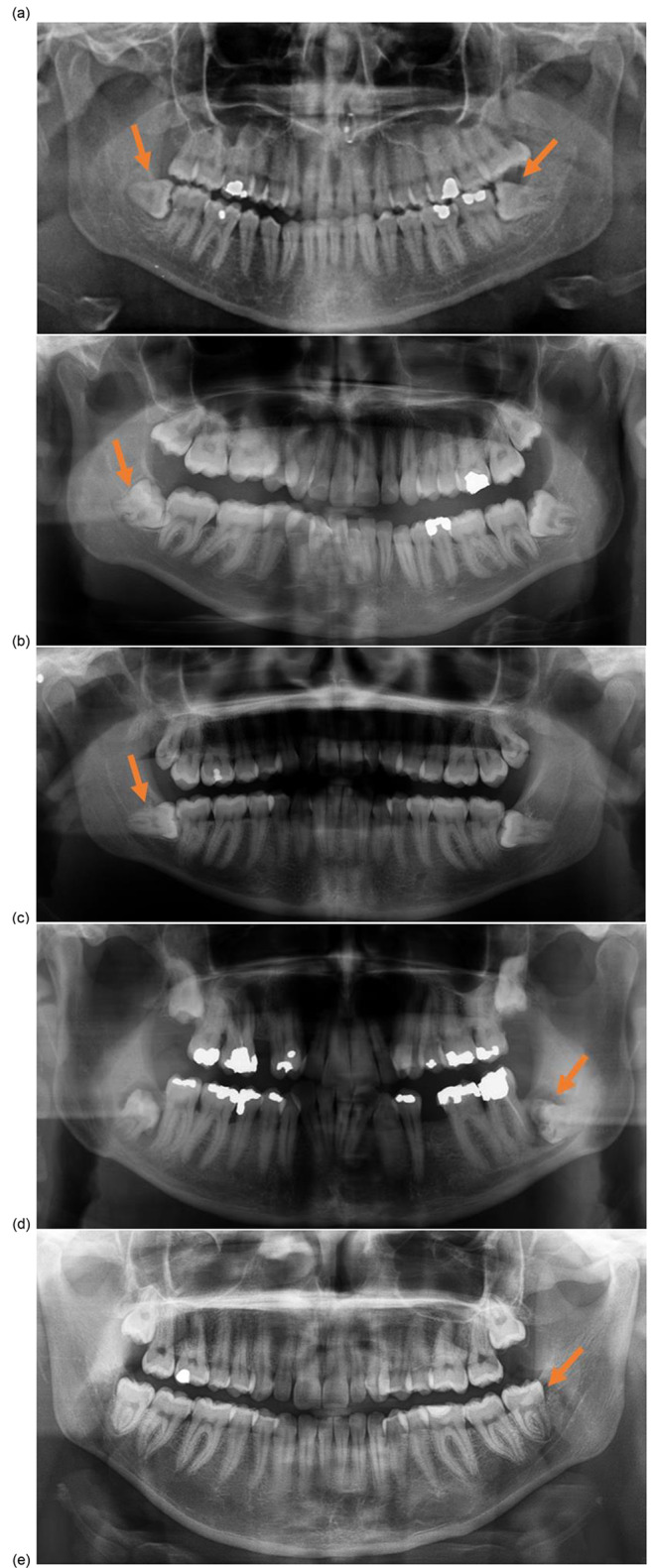
Intra-operative failure where M3M root was removed during the procedure due to increased mobility, marked by arrow

**Fig. 2:**
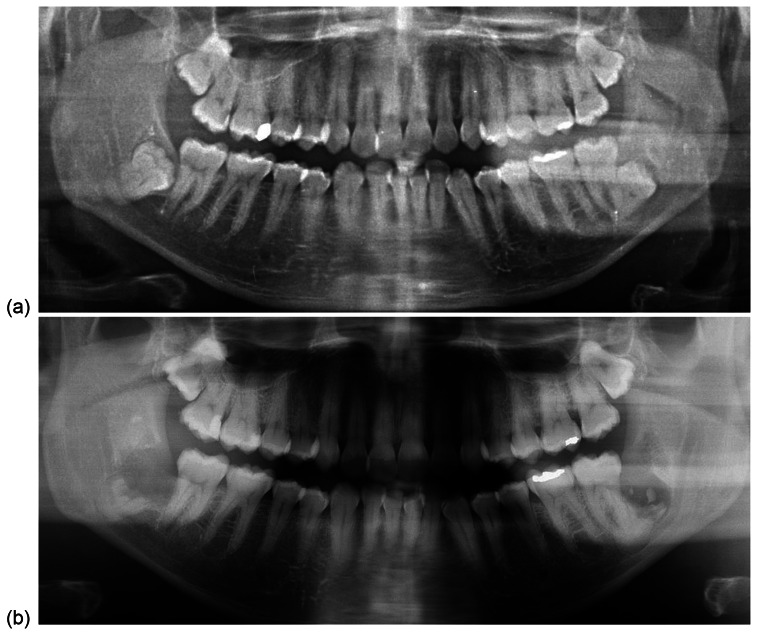
Pre-operative (**a**) and post-operative (**b**) orthopantomograms of Case 1

Intra-operative failure included intra-operative root mobility and the need for root removal (*n* = 5) (Fig. 1) or removal of a single root in a multi-rooted tooth (*n* = 1); and inability to remove enamel fully due to poor access (*n* = 2) (Fig. 2) The case depicted on Fig. 1(d) was associated with significant pus discharge and root mobility conforming the need for removal of the entirety of M3M. M3M associated with intraoperative failure had mesio-angular (*n* = 1), horizontal (*n* = 4) and vertical (*n* = 1) angulation and conical (*n* = 3), short (*n* = 2) and multi-rooted (*n* = 2) root morphology.

The rate of intra-operative failure where M3M root was removed was recorded as 3.6%. No patients in this group suffered temporary or permanent IAN or lingual nerve (LN) damage.

#### Pain/infection/dry socket

Post-operative complications were recorded as pain 14% (*n* = 24), 9% infection (*n* = 15) and dry socket 3.6% (*n* = 6).

Pain was recorded as patient reported and not managed with self-administered analgesia. Optimisation of analgesia advice was provided. Supplementary analgesia was prescribed, if deemed necessary.

Infection was managed for most cases with oral antibiotics (*n* = 8), intravenous antibiotics (*n* = 1) or salt water mouth rinses (*n* = 1). Symptoms settled for all but one who was experiencing recurrent infections, thus, required root removal at 3 months post-surgery.

Dry socket was managed with Alvogyl dressing (*n* = 3), Alvogyl dressing and antibiotics (*n* = 2) and oral antibiotics (*n* = 1). One patient was experiencing persistent pain and had root retrieval at 3 months post-surgery.

#### Nerve injury

Eight patients suffered a nerve injury, including both IAN and LN. Three patients had more than one site affected. A total of nerve injuries was recorded as 12; 9 temporary (2 IAN – 1.2%, 4 LN – 2.4%, 3 site not specified – 1.8%) and 3 permanent (2 LN – 1.2%, 1 IAN – 0.6%). The details of all nerve injury cases are outlined in the Table 6.

**Table 6:** Case details of nerve injury (MA = mesio-angular, TI = transverse, DA = distoangular, VI = vertical, N/R = not recorded, N/A = not applicable)

Pt	Age	Sex	Tooth	Impaction	Reason for surgery	No. high risk signs OPT	CBCT	CBCT signs	IAN position	Nerve injury	Permanent/ Temporary	Other	Follow up
#1	38	M	LR8	MA	Cyst	2	Yes	Significant narrowing and deviation	Lingual	IAN	Permanent	N/A	Discharge 1 yr
#1	38	M	LL8	TI	Cyst	> 3	Yes	Deviation and minimal narrowing, IAN at coronal level	Unknown	LN	Temporary	Lingual retraction	Resolved 5/12
#2	33	F	LL8	DA	Pericoronitis	2	Yes	Significant narrowing and deviation	Lingual	IAN	Temporary	N/A	Discharge
#3	40	F	LL8	MA	Caries in M2M	2	No	Not taken	Not taken	N/R	Temporary	Dry socket	Resolved 2/12
#2	33	F	LR8	DA	Pericoronitis	1	Yes	Significant narrowing and deviation - tear drop	Lingual	N/R	Temporary	N/A	Discharge
#4	25	F	LR8	DA	Pericoronitis	3	Yes	Inter-radicular IAN position	Inter-radicular	N/R	Temporary	N/A	Discharge
#5	30	F	LL8	VI	Pericoronitis	2	Yes	Light contact, no narrowing, no deviation	N/A	LN	Permanent	Lingual retraction	Referral to nerve repair centre – improvement post surgery
#6	40	F	LR8	MA	Pericoronitis	3	Yes	Significant narrowing and deviation	Lingual	LN	Permanent	Altered taste LR8; No lingual retraction	Discharge 1 yr
#7	36	M	LR8	MA	Caries in M2M	1	No	Not taken		LN	Temporary	Root migration	Discharge 2 yrs
#8	47	M	LR8	MA	Cyst	> 3	Yes	Inter-radicular IAN position	Inter-radicular	LN	Temporary	No lingual retraction	Discharge
#8	47	M	LL8	MA	Cyst	3	Yes	Inter-radicular IAN position	Inter-radicular	LN	Temporary	No lingual retraction	Discharge

Below are accounts of patients who suffered a permanent nerve impairment

#### Case 1

A 38-year-old male patient underwent bilateral coronectomies of deeply impacted right and left M3Ms, both associated with a cyst, performed under GA. The right M3M was deeply impacted and mesio-angular (Fig. 2a). The IAN was passing lingual to the apices of M3M, associated with narrowing and deviation. The right M3M demonstrated signs of ankylosis. The left M3M was disto-lingually positioned and deeply impacted with IAN running at the coronal level of the M3M lingually. Access and visibility were described as ‘limited’. A lingual flap was raised and a ‘Howarth’s’ retractor was used for lingual nerve protection of the lower left M3M. Crowns of both M3Ms could not be predicably sectioned and enamel remnants were retained (Fig. 2b). The patient experienced dysaesthesia of right IAN and anaesthesia of left LN. Intra-oral healing was otherwise unremarkable, with full mucosal closure. No evidence of communication or infection were present. Re-operation and removal of retained enamel fragments were considered but thought not to benefit the patient, thus, were not undertaken. The patient was followed up for a duration of 10 months and discharged with persistent right IAN dysaesthesia and fully recovered left LN. Three years passed since the procedure completion. At the time of publication, the patient was not known to experience any issues relating to retained enamel fragments and accepted the status of nerve damage.

#### Case 2

A 30-year-old female underwent a coronectomy of left M3M for chronic pericoronitis. The M3M was vertically impacted. No bony separation between the root and the IAN canal was confirmed on a CBCT. A lingual flap was raised and lingual retraction was used during surgery. Post-operatively, the patient experienced initial paraesthesia and developed complete anaesthesia of left LN within 12 months of the surgery. The patient was referred for LN repair at a specialist unit resulting in successful outcome and improvement of numbness. The patient remains under follow up.

#### Case 3

**Fig. 3:**
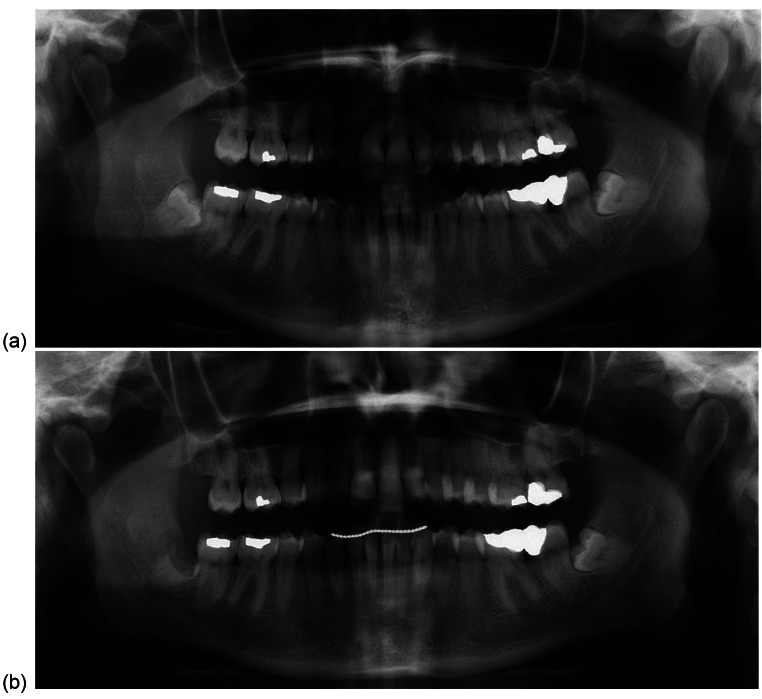
Pre-operative (**a**) and post-operative (**b**) orthopantomograms of Case 3

A 40-year-old male underwent a coronectomy of right M3M for chronic pericoronitis. The M3M was mesioangular demonstrating darkening of roots and deflection of the IAN canal on OPT (Fig. 3). CBCT confirmed lingual position of the IAN canal that was deviated and significantly narrowed. No lingual retraction was used. Post-operatively, the patient experienced paraesthesia of right LN and altered taste. The patient was followed up for 12 months, accepting of the injury as ‘permanent’ and was discharged.

Notably, an enamel fragment was retained mesially (Fig. 3b). Full mucosal healing was achieved and no post-operative infection was recorded. The procedure was performed by a junior staff member, likely conforming a technique resulting in incomplete enamel removal.

There could be a number of theories for LN injury in this case. It is unlikely that retained enamel is the culprit. However, attempts to section the tooth resulting in lingual plate breach could be a factor in mechanical LN injury. No investigations, such as CBCT, were available to confirm this deduction. Finally, a possible mechanism of LN injury also includes a poor IAN block technique under GA.

#### Re-operation

**Fig. 4:**
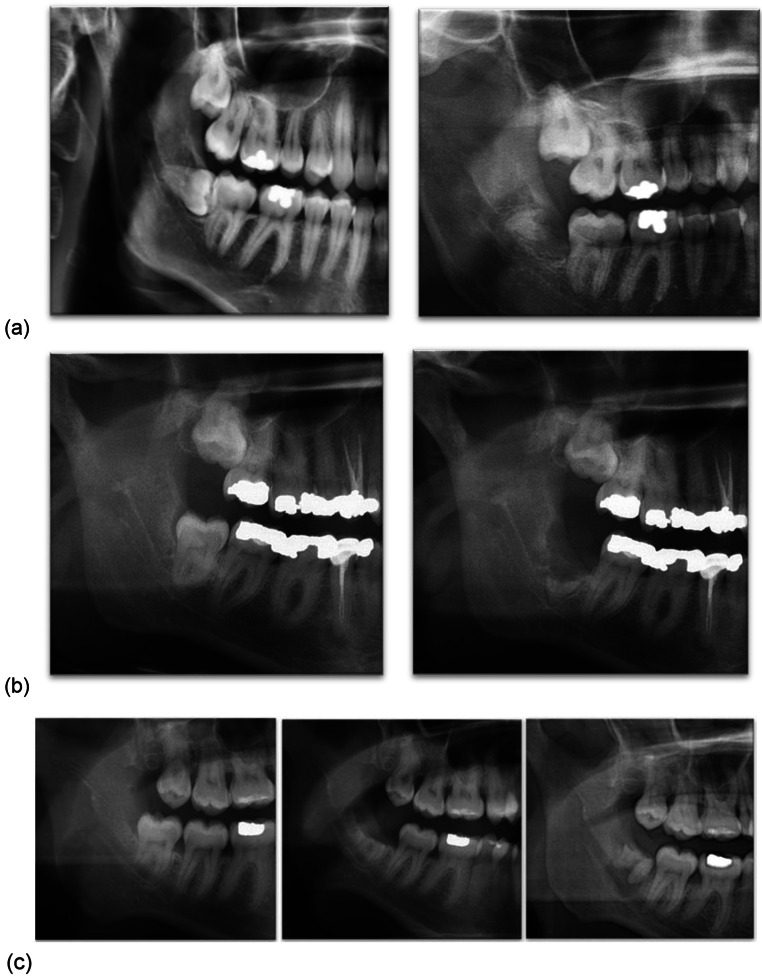
Orthopantomograms of cases requiring re-operation. (**a**) Case 1, (**b**) Case 2 (**c**) Case 3 demonstrating root migration

The reoperation rate was 1.8% as 3 patients required removal of M3M root (Table 7). Two patients underwent root retrieval at 3 months following coronectomy without the evidence of root migration (Fig. 4(a) Case 1; (b) Case 2). Both patients recovered fully and did not experience temporary or permanent IAN injury. One patient required surgical removal of M3M retained roots 2 years after coronectomy. Root migration was noted on radiographs, highlighting successful outcome and the intent of the procedure (Fig. 4(c) Case 3). No post-operative IAN injury was recorded.

**Table 7:** Clinical details of re-operation cases. (F = female, N/R = no relevant; HI = horizontal, DA = distoangular, VI = vertical; ST = specialty trainee, SPD = specialty dentist)

	Age, Sex	Medical/ Social history	Impaction	Pulpal status	Root morphology	Operator grade	Anaesthetic type	Time until re-operation	Reason for re-operation
1	27, F	N/R	HI	Sound	Conical	ST	LA	3/12	Post op infection, persistent soreness, lump throat – rule out cause
2	39, F	N/R	DA	Sound	Two rooted	SPD	GA	3/12	Persistent pain, lump, OPT PDL widening
3	29, F	Smoker	V	Caries into outer dentine	Two rooted	ST	LA	+ 2 yrs	Recurrent infections, root migration

### Follow up protocol

Overall, 15% of post-operative coronectomy cases presented with an acute issue within 1–2 weeks following the surgery. 82% of coronectomy cases had a scheduled follow up appointment. 19% of cases presented prior the scheduled appointment. Out of those who did not have a scheduled follow up, 7% presented acutely.

### Success of coronectomy

**Fig. 5:**
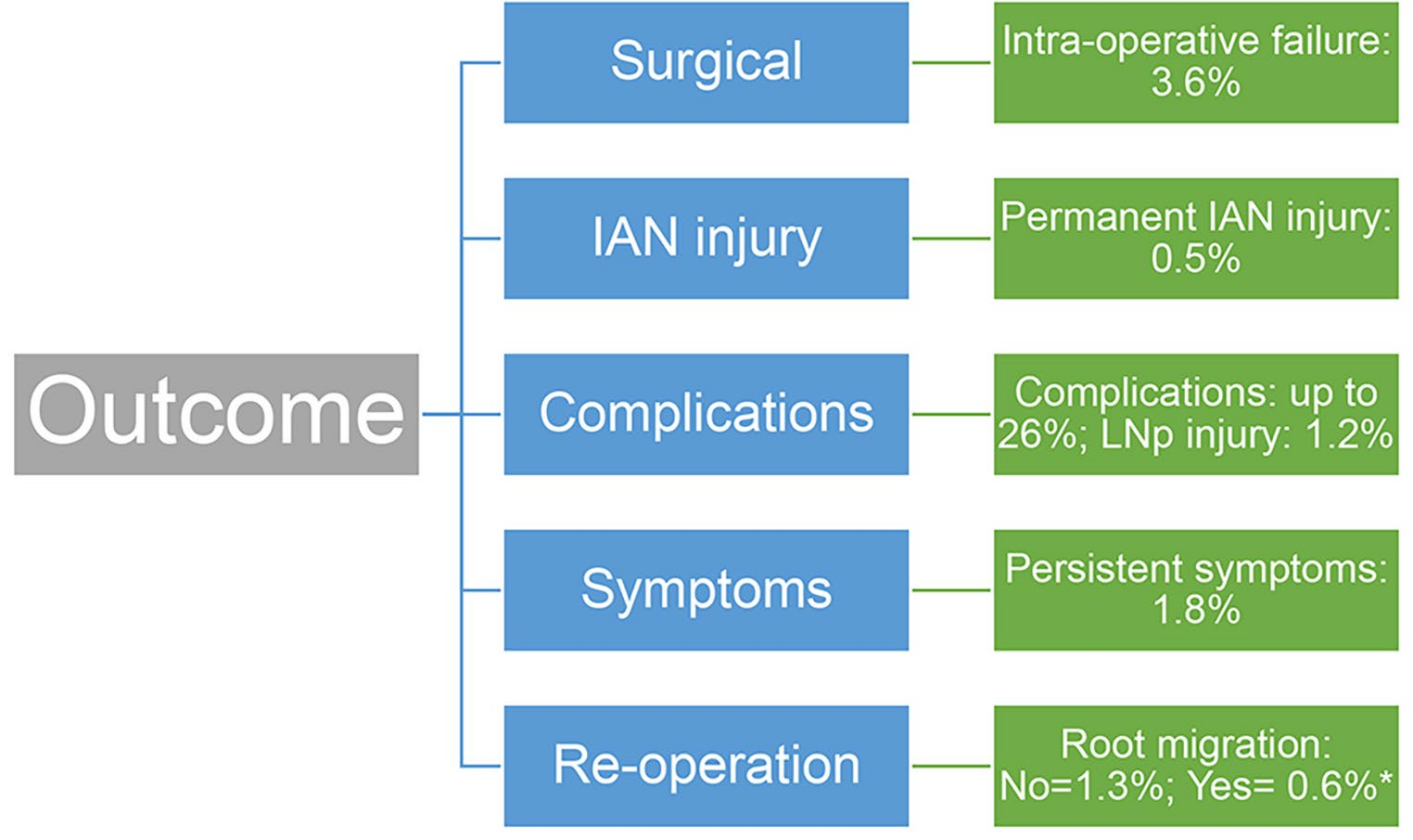
Coronectomy success assessment tool. Success rate = 100% - total % of complication (* = discounted from total complication rate)

The outcome of coronectomy can be determined as a factor of surgical outcome, presence or absence of permanent IAN injury, persistent symptoms or any other long-standing complications (such as LN injury), and the need for re-operation considering root migration status (Fig. 5). The flowchart accounts for total rate of complication, and success is considered in the absence of these (Success rate = 100% - total % of complication).

The complication rates in our study are applied in the flowchart in Fig. 5. In our instance, the rate of persistent symptoms and re-operation rates represent the same cases. Re-operation where root migration was evident carries a low risk of IAN injury. Cases falling into this group are given a successful outcome, thus, were discounted from the total complication rate (*). In this study, the successful outcome of coronectomy was 93%.

## Discussion

### Success of coronectomy

The outcomes of the study were compared against published studies (Table 8). We propose the use of our coronectomy success assessment tool (Fig. 5) to be implemented in practice and in the future studies. It offers a holistic approach to evaluation of coronectomy outcome including consideration of persistent symptoms and any long-lasting complications that impact the patient’s quality of life.

**Table 8:** A review of the outcomes and complications of coronectomy studies. (RCS = retrospective case series, RCC = retrospective case control study, PCC = prospective case control study, PCHS = prospective cohort study, RCGS = retrospective cohort study, RCT = randomised control trial; T = temporary, P = permanent)

Author	Country	Study design	No. cases	Intra-op failure (%)	Pain (%)	Dry socket (%)	Infection (%)	tIAN injury (%)	pIAN injury (%)	pLN injury (%)	tLN injury (%)	Re operation (%)
Frenkel et al. 2014 [10]	Israel	RCS	185	.	8.6		.	0	0	0.5	0	3.2
Hamad 2023 [11]	Iraq	RCC	220	.	.	4.5	6.4	0	0.5	.	.	3.2
Kang et al. 2019 [12]	China	PCC	55	.	.	1.82			0	.	.	9
Kouwenberg et al. 2015 [13]	Netherlands	PCHS	151	.	.	.	.	0	0	0	0	11.3
Leung and Cheung, 2009 [14]	Hong Kong	RCT	171	9.4	41.9	0	5.8	0	0	.	.	0.6
Leung et al. 2016 [15]	Hong Kong	PCHS	612	.	31.2	0.16	2.9	0	0	0	0	2.1
Monaco et al. 2017 [16]	Italy	PCHS	116	.	9	4	.	0	0	0	0	6
Pedersen et al. 2018 [17]	Denmark	PCHS	231	.	.	.	11.7	1.3	0.87	.	2.2	3.5
Pitros et al. 2019 [18]	UK	RCHS	133	.	19.8	14.6	13.7	4.3	3.5	.	.	0.8
Renton et al. 2005 [3]	UK	RCT	94	36	13.8	12.1	5.2	8	0	0	0	0
Al-Raisi et al. 2022 [19]	UK	RCHS	187	.	.	2.1	4.8	0.5	0.5	.	0.5	1.6
Hanato et al. 2009 [20]	Japan	PCC	102	5.06	18.63	1.96	0.98	1	0	0	0	4.9

### Assessment strategies

Inter-observer variability for detection of high-risk signs on OPT is well documented [21]. Surgeons with less experience are more likely to be cautious and over-diagnose high risk signs on conventional imaging. CBCT can predictably determine the position of IAN in relation to M3M roots. Although, CBCT is offered, patients may decline additional imaging due to added cost or delay to treatment. According to SEDENTEX guidelines, once a decision has been made to proceed with coronectomy on a plain film radiograph, CBCT is not necessary [22]. However, the value of CBCT is highlighted by Matzen et al., where it can influence the treatment plan for 12% of cases from surgical removal to coronectomy and 6% from coronectomy to surgical removal [5]. 30% of high risk M3M on conventional imaging are distant on CBCT [4]. For these cases, surgical removal is preferred.

Pitros et al. discussed the cost-effectiveness of coronectomy versus surgical removal [8]. CBCT adds treatment cost to the patient or the health service. However, if a decision to surgical remove the M3M is made as a direct result of the scan, the overall cost of M3M surgery is lowered, avoiding the potential second surgery cost.

In our study, we note that a small number of coronectomies were completed on patients with low risk M3M, objectively identified on conventional and CBCT imaging. For example, in Fig. 1(a), while there are high-risk signs left M3M, there is bony separation for right M3M, rendering right M3M low risk. In our experience, the discussion of an increased IAN injury risk increases patients’ fear of neurological deficit and makes coronectomy the ‘safer option’. Given this, some patients opt to have coronectomy for the ‘lower’ risk M3M also, despite the surgeon’s recommendation, particularly if this if considered for contralateral M3M. In this study, patients were fully informed about risks and benefits of treatment offered. With capacity to consent, patients’ material values were respected, as coronectomy is a valid management strategy of M3Ms.

Authors promote the use of CBCT for enhanced decision-making for wisdom teeth surgery to guide patients for most appropriate treatment options, particularly if low risk is determined.

### Complication: pain

Post-operative pain not managed by self-administered analgesia was the highest short-term complication recorded in the study at 14%. Similar post-operative pain scores were described by Renton et al. (13.8%) [3]. As in our retrospective study, most of studies in the literature did not use visual analogue scale (VAS) to assess pain levels [3, 10]. A clear limitation of objective pain assessment is highlighted. Comparative results were published by Pitros et al. (19.8%) and Hanato et al. (18.63%), however, VAS was only used in the latter study [18, 20].

In a randomised control trial, Leung and Cheng recorded highest pain score, with 41.9% coronectomy patients reporting pain one week post-surgery, significantly lower (*n* = 0.005) than the pain following surgical wisdom teeth removal (57.3%) [14], sharing findings with Renton et al. [3], but contrary to Hanato et al. [20] who reported higher pain levels post-coronectomy (18.63% vs. 6.78%).

### Complication: dry socket

The incidence of dry socket is reported as low as 0.16% in a large prospective cohort study of 612 coronectomy cases [15] and as high as 14.6% in a retrospective cohort study of 133 cases [18]. Dry socket is the most common complication of wisdom teeth surgery, and its incidence is similar in both surgical removal and coronectomies [8]. ‘Dry socket’ symptoms in coronectomy patients should be managed traditionally with irrigation and careful placement of ‘Alveogyl’ [23]. Recurrent symptoms may indicate a need for consideration of re-operation. In our study, no patients presenting with dry socket were required further intervention beyond socket dressing.

### Complication: infection

Infection rates of 9% presented in this study were average compared to the literature. Lower infection rates as low as 0.98% and 2.9% in studies by Hanato et al. [20] and Leung & Cheung [15], respectively. While Pendersen et al. [17] and Pitros et al. [18] reported infection rates as high as 11.7% and 13.7% respectively.

The limitation of our study was lack of standardisation of post-operative infection assessment. Infection may have been over-diagnosed, presenting as post-operative pain and swelling in patients presenting acutely. Differentiation between true post-operative infection and normal post-operative levels of pain and swelling may be difficult by junior staff who are the front line of the emergency departments. Pre-cautionary antibiotics may have been given to patients in an acute setting to minimise potential risk of early failure and need for re-operation without root migration. Only one patient who had an infection had a root removal within 3 months of the first surgery. This suggests that antibiotic therapy is effective in settling down infection during early stages of post-operative healing.

### Complication: nerve injury

The goal of coronectomy is to treat high risk M3M and minimises the risk of IAN injury, however, it does not fully nullify this complication. In our study, one patient suffered a permanent IAN disturbance (0.6%) following a coronectomy for a deeply impacted M3M associated with a cyst.

At least 1.2% cases suffered temporary IAN injury, however, this may be mis-reported due to lack of site specification of 1.8% cases of transient nerve deficit in the study. In the literature several studies report 0% permanent IAN injury, whereas other authors found the rates varying from 0.5 to 3.5% [11, 17, 18]. In a randomised control trial, Renton et al. described a complete recovery in 8% of cases with initial transient IAN dysesthesia. In the study no patients had temporary or permanent lingual nerve disturbance [3] (Table 8).

Transient LN injury was noted for 2.4%, whereas two patients suffered a permanent LN injury (1.2%) with final diagnosis of such at 12 months post- surgery. Both patients appeared to have low surgical complexity. Frenkel et al. [10] reported 0.5% sustaining a permanent LN paranaesthesia. The most common cause for LN paraesthesia is lingual retraction [24] and is an attributable cause of the LN injury in one of our cases of complete anaesthesia *(Nerve Injury Case 2)*. Following one year follow-up, the patient underwent lingual nerve repair at a specialist nerve repair centre resulting in an improvement and a result acceptable by the patient (complete anaesthesia to paraesthesia). A referral to specialist centre for lingual nerve repair should be considered ideally within one month for optimal prognosis [25]. It is understood, however, that such centres may not be available for majority of practitioners worldwide and only select cases may be suitable for the procedure. A direct cause of LN could not be identified for the other case of permanent LN paraesthesia (*Nerve Injury Case 3*). Mechanisms for injury that were disputed include mechanical breach of lingual plate with surgical drill or IAN block under general anaesthetic. It can be speculated that deep coronal section can sever the lingual plate or superficially placed LN. Care should be applied during this step, favouring incomplete crown sectioning approach described by Gleeson et al. [26].

Coronectomy does not protect against lingual nerve injury, which, it can be very debilitating affecting quality of life [27]. Permanent lingual nerve injury is considered as a serious complication of M3M surgery, thus, should be considered as an unsuccessful outcome in provision of treatment, leaving the patient with a persistent complication. Lingual retraction was identified as a causative factor in one case of the permanent LN injury. Operator experience, poor technique and assistant experience or surgical case complexity may be factors involved leading to permanent LN injury. Routine use of lingual retraction for coronectomy is not recommended.

Patients with a history of more than 12 months altered sensation, whose nerve disturbance did not affect their daily activities were considered to have permanent nerve injury and were discharged from follow-up care.

It is unlikely that complete resolution of nerve sensation will recur if the sensory deficit is still present at nine months [28], however full nerve recovery may take up to 24 months. The 12-month follow-up period for nerve injury patients may be considered as a limitation of this study as it may be speculated that some of nerve injuries included in this study recovered, changed character, or stayed the same.

### Re-operation

Re-operation rates in our study can be considered as low when compared to the literature, ranging from 0.5 to 11.8% (Table 8). Renton et al. [3] demonstrated 0% of re-operation rates. The authors, however, reported 36% of intra-operative removal of loose roots on the assumption of lost tooth vitality, therefore, negating the potential in increased failure.

There are concerns that M3M with deep decay or resorptive defects may be a factor in failure of coronectomies and percipitate the need for early re-intervention. In our study, out of 13 patients with dentine caries, only one patient required re-operation at 3 months following initial surgery. One patient with caries extending beyond the outer third but not into pulp was lost to follow up, however, did not re-present with symptoms 3 years since the surgery. Two patients who had a coronectomy on a M3M with internal resorption had a successful outcome. In a case series, Patel et al. [29] demonstrated 14 cases with dentine caries and seven cases of internal resorption in M3M treated with a coronectomy were asymptomatic at one year follow up. The results sound promising and are suggestive that coronectomy offers a favourable alternative to surgical removal in high risk M3M and should not be disregarded. Appropriate discussion with the patient about potential risk of failure and early re-operation should be undertaken.

### Reasons for failure

Leung and Cheung (2009) report intra-operative failure rates as 9.4%, whereas Renton et al. (2005) demonstrated highest failure rates of 38%. In our study, we recorded 5.4% intra-operative failure rates, similar to Hanato et al. (4.9%) [20]. We believe that our results are likely to be under-reported due to recording and coding surgical procedure variability (Coronectomy vs. Surgical removal of Wisdom Tooth) and retrospective design of the study, making factual capture challenging.

Intra-operative failure carries a heightened risk of IAN injury. None of the patients in our study who had M3M roots removed intra-operatively had transient or permanent IAN injury post-operatively. It is of essence to fully inform the patient about this potential risk and its implications as part of the consent process. In the randomised control trial by Renton et al. 8.3% of ‘failed coronectomy’ cases suffered IAN disturbance, compared to 0% in successful coronectomies in the same study. High risks for increased intra-operative mobility were female, conical root formation and narrowing of roots within the canal [3]. Leung and Cheung reported 9.4% of intra-operative failure in their study, with one case left with a permanent IAN deficit. The authors found no significant risk factors associated with coronectomy failure (age, sex, root morphology, impaction) [15].

Intra-operative failure may be attributed to poor case selection with relation to root morphology, as most frequently associated with short and conical roots. Horizontally impacted wisdom teeth are technically difficult to remove coronal tissues 3 mm below the crestal bone. Conical roots are at the increased risk of intra-operative mobility following decorontation [3]. Aside from IAN relationship, attention should be paid to root angulation and root morphology, and operator grade when considering coronectomy as a M3M management strategy.

### Authors comments

The Mid-Yorkshire Teaching NHS Trust is a tertiary unit accepting referrals from primary and secondary care. The Adult Oral Health Survey 2021 highlighted that 64% of adults have moderate to high levels of dental anxiety in the United Kingdom [30]. Patients treated in this study had moderate to high Modified Dental Anxiety Scale (MDAS) scores [31] upon referral to the service. Given the surgical nature of wisdom teeth treatment, majority of the procedures in this study were completed under general anaesthetic. The authors believe that routine coronectomy can be safely and predictably carried out under local anaesthetic or with the aid of conscious sedation.

## Conclusion

Coronectomy offers a safe and effective treatment strategy in management of selected high risk M3M with a body of evidence for its use. The success of coronectomy can be as high as 93% determined as a factor of surgical outcome, presence or absence of permanent IAN injury, persistent symptoms or any other long-standing complications, and the need for re-operation giving consideration to the root migration status. The treatment outcomes can be further improved with careful case selection and adjustment of surgical technique, including assessment of root morphology, incomplete crown sectioning technique and avoidance of lingual retraction. The coronectomy success assessment tool described is a valuable aid in reporting coronectomy outcomes in practice and in further studies.

## Data Availability

No datasets were generated or analysed during the current study.
